# Challenges Posed by Tick-Borne Rickettsiae: Eco-Epidemiology and Public Health Implications

**DOI:** 10.3389/fpubh.2015.00055

**Published:** 2015-04-21

**Authors:** Marina E. Eremeeva, Gregory A. Dasch

**Affiliations:** ^1^Jiann-Ping Hsu College of Public Health, Georgia Southern University, Statesboro, GA, USA; ^2^Rickettsial Zoonoses Branch, Division of Vector-Borne Diseases, Centers for Disease Control and Prevention, Atlanta, GA, USA

**Keywords:** *Rickettsia*, spotted fever rickettsioses, ticks, co-feeding transmission, transovarial maintenance, acquisition feeding, eco-epidemiology, molecular epidemiology

## Abstract

Rickettsiae are obligately intracellular bacteria that are transmitted to vertebrates by a variety of arthropod vectors, primarily by fleas and ticks. Once transmitted or experimentally inoculated into susceptible mammals, some rickettsiae may cause febrile illness of different morbidity and mortality, and which can manifest with different types of exhanthems in humans. However, most rickettsiae circulate in diverse sylvatic or peridomestic reservoirs without having obvious impacts on their vertebrate hosts or affecting humans. We have analyzed the key features of tick-borne maintenance of rickettsiae, which may provide a deeper basis for understanding those complex invertebrate interactions and strategies that have permitted survival and circulation of divergent rickettsiae in nature. Rickettsiae are found in association with a wide range of hard and soft ticks, which feed on very different species of large and small animals. Maintenance of rickettsiae in these vector systems is driven by both vertical and horizontal transmission strategies, but some species of *Rickettsia* are also known to cause detrimental effects on their arthropod vectors. Contrary to common belief, the role of vertebrate animal hosts in maintenance of rickettsiae is very incompletely understood. Some clearly play only the essential role of providing a blood meal to the tick while other hosts may supply crucial supplemental functions for effective agent transmission by the vectors. This review summarizes the importance of some recent findings with known and new vectors that afford an improved understanding of the eco-epidemiology of rickettsiae; the public health implications of that information for rickettsial diseases are also described. Special attention is paid to the co-circulation of different species and genotypes of rickettsiae within the same endemic areas and how these observations may influence, correctly or incorrectly, trends, and conclusions drawn from the surveillance of rickettsial diseases in humans.

## Introduction

Rickettsiae are obligately intracellular bacteria with complex life cycles that are dependent upon certain animals, mostly vertebrate mammals, and also include reptiles and birds, and diverse arthropods for their survival. Among them, arguably, the tick-borne agents are the best studied group. These certainly are the most important group as regards veterinary, wildlife, and human diseases because they transmit most of the large spotted fever group of rickettsiae (SFGR) that cause disease in humans. These currently include over 25 formally recognized species and an ever growing number of unnamed and non-cultivated genotypes, which are still poorly characterized (1). However, some of these new rickettsiae have proven to be causes of emerging human diseases; they are often first recognized from their associations with different animals and their ectoparasites and only later detected in clinical specimens and associated with specific diseases. Most frequently, the detection of new rickettsiae has occurred by detailed examination of different species of ticks so this distribution may well be biased by that methodology, rather than reflecting the true abundance and distribution of rickettsial lineages in nature (2).

The majority of tick-borne rickettsiae belong to the core classic spotted fever group (3). This bias may, in fact, just reflect the focus of medical and veterinary studies on ticks and the use of classic procedures and molecular procedures that work well only with this subgroup, rather than the diversity of rickettsiae found in ticks, let alone the full range of arthropods known to harbor rickettsiae. Historically, SFGR were referred to as the agents of a group of endemic rickettsioses, implying their focal distribution and limited associations with specific ecological settings (4). The advent of molecular tools and their commonplace application to investigation of associations of rickettsiae with their animal and invertebrate hosts has significantly changed this picture and our understanding of these variable associations. In 1982, Nyven Marchette wrote that the ecological relationships of rickettsiae for the most part are known or at least amenable to investigation (5). In his extended monograph, Dr. Marchette thoroughly summarized the facts about known or reported associations of spotted fever group rickettsiae with different tick species but sadly, many aspects of these associations still remain unexplored more than 40 years later (6), possibly due in part to overreliance on tools developed in the “molecular era.”

Typically rickettsioses are described as zoonotic diseases, although, the term “zoonosis” is rather inaccurate and loosely used in this context compared to its primary definition as a disease that normally exists in animals but can infect humans. Indeed, two contemporary unresolved issues regarding rickettsioses and public health are highlighted with this problematic usage: (a) How large a role do animal hosts play in the life cycle of rickettsiae, aside from their essential role as a blood source for their tick hosts? Do true zoonotic rickettsial infections really occur? and (b) What routes of infection of animals and humans are most important in the acquisition of spotted fever rickettsioses? Is the feeding and salivating tick or the infected tick itself all that is important in transmission? – Are infected excreta of ticks important source of infection and do these infectious powders originate directly from the animal hosts of ticks post feeding, from a tick contaminated environment or only on a host during the acquisition of the blood meal?

Here, we evaluate and reflect on the contemporary knowledge and understanding of *Rickettsia*–host and *Rickettsia*–vector interactions in the greater context of recent findings on invertebrate immunity, microbial communities of arthropods, and especially on vector associations with various primary and secondary endosymbionts. We attempt to identify key gaps in our understanding of the eco-epidemiology of SFG rickettsioses and their importance for understanding the epidemiology of human diseases caused by these microorganisms. Finally, we discuss how these varied biological associations in ticks may influence outbreaks of rickettsial infections, their implications for epidemiological investigations, and provide relevant public health recommendations.

To paraphrase George Santayana and take his advice (7), we will first review current dogma and then revisit the conclusions of the pre-molecular era in order to suggest what is still needed from contemporary investigations. In particular, we examine the gaps in the ways that our increasingly powerful contemporary molecular tools are being used to address these questions.

## Recent Shifts in Dogma about the Epidemiology of Classic Rickettsial Tick-Borne Diseases

The incidence of tick-borne rickettsial diseases is currently going through its second pronounced increase in the last 40 years. Since 1970s, four endemic rickettsioses Rocky Mountain spotted fever (RMSF), Mediterranean spotted fever (MSF), North Asian tick typhus (NATT), and Queensland tick typhus (QTT), have been on a continuous increase (4). The incidence of Japanese spotted fever has also increased steadily since its discovery in the mid-1980s (8). It is possible that other tick-borne rickettsial infections have shown similar increases but only these more common and severe diseases have much useful, albeit based on the limits imposed by contemporary views, disease surveillance information. Ecological factors, particularly those driven by climate change, surveillance methodologies, and human population increases and behavioral changes (recreation, association with nature) may all be contributing factors to this phenomenon (9, 10). Elevated attention to this increase and the advent and adaptation of new molecular tools used for field and laboratory studies in the 1990s, complemented by increased funding support for these studies has opened Pandora’s Box. The discovery and description of novel nosological entities caused by previously unknown spotted fever group rickettsiae has continued unabated since then (1, 11). Consequently, to a degree, the traditional views of tick-borne rickettsioses as endemic diseases with largely focal distributions and limited host and geographic ranges, predetermined seasonality and defined tick associations became obsolete or at least very incomplete (12). This expansion of our awareness about the existence of other rickettsial agents with varied clinical and epidemiological attributes has been thoroughly reviewed but it has presented new challenges to the medical and public health communities (1). This paradigm shift is due to the fact that numerous rickettsiae of unknown to variable degrees of pathogenicity for humans co-circulate in overlapping geographic regions and may even be found in the same tick species. The details of these findings and those vector and geographic associations have been reviewed and summarized by several authors (1, 11).

Within the limits of current knowledge, RMSF is endemic throughout the Americas and MSF is endemic through southern Europe and Africa to the Asian subcontinent. These are still the most prevalent spotted fever rickettsioses requiring medical attention and stand out for their morbidity and mortality. Thus, these are still the priority agents for surveillance of reportable rickettsial diseases in the corresponding countries where they are found. At least eight human rickettsial pathogens circulate in ticks in different and often overlapping parts of Eurasia (including *R. conorii*, *R. massiliae*, *R. slovaca*, *R. raoultii*, *R. sibirica*, *R. mongolotimonae*, *R. helvetica*, *R. rioja*, and possibly others), at least four in Africa (*R. africae*, *R. conorii*, *R. massiliae*, and *R. aeschlimanni*), four in Australia (*R. australis*, *R. honei*, and *R. honei* subsp. *marmionii* and possibly *R. gravesii*), and several in the Americas (*R. rickettsii*, *R. parkeri*, and *R. massiliae*). *Rickettsia amblyommii* and its closely relatives are highly prevalent SFG rickettsiae in the aggressive human-biting tick *Amblyomma americanum* in the USA and *Amblyomma* spp. in Central and South America; their pathogenicity for humans is widely speculated but not yet clearly demonstrated (1, 13–15). Some rickettsioses are known to cause only a handful of cases but it remains to be determined whether their impact will always be small because of their low pathogenicity or low vector carriage or transmission potential or these numbers are just a reflection of their being discovered only recently and diagnostic assays are still insufficiently specific to determine which agent is causing an infection. However, even low pathogenicity agents may contribute to the apparent overall increased incidence of rickettsial diseases in the world because cross-reactive serological tests are still the primary means for diagnosing rickettsioses. Many of these “cases” may, in fact, reflect an unrelated exposure to a tick bearing a *Rickettsia* agent that caused an immune response rather than the occurrence of a rickettsiosis. This fact led to the change in national reporting of RMSF cases in the United States to their classification as spotted fever rickettsioses. Consequently, a central issue for public health remains: whether or how our greatly expanded knowledge on the current temporal and spatial distribution of rickettsial agents will have any effect on medical practice and the diagnosis of rickettsioses. This is particularly true for underdeveloped regions where rickettsioses may occur in the same locations as high impact diseases such as malaria, leptospirosis, arboviral infections, and other diseases presenting initially with a fever, headache, and/or rash – the most prevalent manifestations of rickettsioses. It is more likely that a better understanding of the complexity of the eco-epidemiology of rickettsial agents with shared vectors and animal hosts will be useful primarily for improved modeling of the life cycles of these agents, for improving our surveillance and outbreak response tools for rickettsioses, and for establishing cost-effective targeted control efforts for the primary problematic agents or their vectors. Whether this long-range strategy will be of greater benefit to society by reducing the disease burden from nature than educational efforts to ensure proper clinical recognition of the affected individuals, or by developing much better therapeutic regimens based on bactericidal antibiotics to reduce the burden of hospitalization, long-term sequelae, or fatal infections is currently unknown.

## Basic Concepts about Rickettsiae and Rickettsial Ecology

Two basic concepts about tick-borne rickettsial ecology originated in the seminal observations made by Ricketts early in the twentieth century who hypothesized that the agent of RMSF, *R. rickettsii*, is maintained in nature in a continuous cycle between infected ticks and one or several of the host animals parasitized by *Dermacentor andersoni* (16, 17); he also speculated that hereditary transmission of *R. rickettsii* in ticks might occur on a limited scale (18). At that time, he conducted limited laboratory experiments and established the susceptibility to *R. rickettsii* of several small animals, including the ground squirrel, rock-squirrel, chipmunk, and woodchuck. He was able to demonstrate that ticks feeding on these hosts did acquire the infectious agent and could subsequently transmit it to guinea pigs. These findings led Ricketts to the hypothesis that new lines of infected ticks (2 years are required for a complete life cycle for this tick) were started each season by simultaneous feeding of different stages of infected and uninfected ticks on susceptible host animals, primarily rodents, rabbits, and hares. However, the extent to which infections persist in the host animal, the relative importance of different hosts for tick maintenance – especially for transmission and acquisition of *R. rickettsii* by different stages of ticks, and the means and efficiency of transfer of rickettsiae from an infected to an uninfected tick were all uncertain and are still open questions today. Thus, these remain as unknown or semi-quantitative variables in modern attempts to model the dynamics of this maintenance-transmission system (19, 20).

Subsequently, many different animal species have been qualitatively associated with maintenance and circulation of *R. rickettsii* in nature either by direct isolation of rickettsiae from tissues or by demonstrating their seroconversion by different immunoassays following needle or tick inoculation of the agent or exposures in nature [reviewed in Ref. (21, 22)]. Some major questions remain unanswered from this work (Table 1): (1) do these animals experience clinical disease or rickettsemia or any agent replication during rickettsial infection; do these reservoirs serve as sources of outbreaks of human disease, and which are the most important; (2) do all of these animals serve as sources of infection for various ticks vectoring RMSF to humans and other animals, and (3) do these animals serve primarily as hosts for feeding of infected and non-infected ticks and a site for facilitating exchange of the pathogen between different ticks and tick life stages or are they otherwise dead end hosts like humans? Similarly, whether the known different tick vectors of RMSF, *D. variabilis*, *D. andersoni*, *Amblyomma* tick species, *Haemaphysalis leporispalustris*, and *Rhipicephalus sanguineus* differ markedly in their abilities and specific mechanisms used to sustain rickettsial populations in nature is still unknown. However, *R. rickettsii* itself has clearly diverged genetically in association with these different vector species and geographic regions (23–25).

**Table 1:** **Variable effects of *Rickettsia* observed in different host animals**.

Rickettsia species, isolate	Associated tick species	Animal species	Observed effects in the source animal	Records of isolations (source)	Reference
*R. rickettsii*, *Microtus* agent B14009^a^	*D. variabilis*^b^	Meadow mouse, *Microtus pennsylvanicus*	None (apparently healthy)	Yes (brain, spleen, and liver)	(26)
*R. rickettsii*, Mp23, Mp40, Pit1	*D. variabilis*^b^	Wild mice, *Peromyscus leucopus*, *Pitymys pinetorum*	Tissue persistence; seroconversion	Yes (liver and spleen)	(27)
*R. rickettsii*, Di6	*D. variabilis*^b^	Opossum, *Didelphis marsupials virginiana*	Tissue persistence; low level seroconversion	Yes (liver and spleen)	(27)
*R. rickettsii*, Rab1	*D. variabilis*^b^	Eastern cottontail rabbit, *Sylvagus floridans*	Tissue persistence; seroconversion	Yes (liver and spleen)	(27, 28)
*R. rickettsii*, Si7	*D. variabilis*^b^	Cotton rat, *Sigmodon hispidus*	Tissue persistence; low level seroconversion	Yes (liver and spleen)	(27)
*R. rickettsii*, Sheila Smith	N/A	Cotton rat, *Sigmodon hispidus*	Tissue persistence; short-term rickettsiemia; low level seroconversion	Yes (blood)^b^	(29)
*R. rickettsii*, Sawtooth	*D. andersoni*	Snowshoe hare, *Lepus americanus*	Rickettsiemia (exp)	Yes (xenodiagnosis)	(22, 30)
*R. rickettsii*, Sawtooth	*D. andersoni*	Golden-mantled ground squirrel, *Citellus lateralis tescorum*	Rickettsiemia (exp)	Yes (xenodiagnosis)	(22, 30)
*R. rickettsii*, Sawtooth	*D. andersoni*	Chipmunks, *Eutamias amoenus*	Rickettsiemia (exp)	Not reported	(22, 30)
*R. rickettsii*, Sawtooth	*D. andersoni*	Columbian ground squirrel, *Urocitellus columbianus*	Rickettsiemia (exp)	Yes (xenodiagnosis)	(22, 30)
*R. rickettsii*, Sawtooth	*D. andersoni*	Meadow mice, *Microtus* spp.	Rickettsiemia (exp)	Yes (xenodiagnosis)	(22, 30)
*R. rickettsii*, Sawtooth	*D. andersoni*	Bushy-tailed woodrat, *Neotoma cinerea*	Seroconversion	No	(22, 30)
*R. rickettsii*, Taiaçu	*A. cajennense*	Capybara, *Hydrochoreus hydrochaeris*	Seroconversion; rickettsiemia (exp) afebrile	Yes (xenodiagnosis)	(31)
*R. rickettsii*, ITU	*A. cajennense*	Capybara, *Hydrochoreus hydrochaeris*	Seroconversion	No	(32)
*R. rickettsii*, Taiaçu	*A. cajennense*	Opossum, *Didelphis aurita*	Rickettsiemia (exp) asymptomatic; no macro or micro pathological abnormalities	Yes (xenodiagnosis)	(33)
*R. rickettsii*, Taiaçu	*Rh. sanguineus*	Dog, *Canis familiaris*	Rickettsiemia (exp)	Yes (xenodiagnosis)	(34)
*R. rickettsii*, Sawtooth	*Rh. sanguineus* (presumably North America)	Dog, *Canis familiaris*	Rickettsiemia (exp); seroconversion	Yes (cell culture)	(35)
*R. rickettsii*, Wachsmuth	*Rh. sanguineus* (presumably North America)	Dog, *Canis familiaris*	Rickettsiemia (exp); seroconversion	Yes (xenodiagnoses)	(35)
*R. rhipicephali*, 3-7-♀6	*Rh. sanguineus* (presumably North America)	Dog, *Canis familiaris*	Seroconversion	No	(35)
*R. montanensis*	*Rh. sanguineus* (presumably North America)	Dog, *Canis familiaris*	Seroconversion	Not tested	(35)
*R. parkeri*	*A. maculatum*	Cattle	Seroconversion	No	(36)
*R. parkeri*, Portsmouth	*A. maculatum*	Cotton rat, *Sigmodon hispidus*	Short-term rickettsiemia; seroconversion	Yes, re-isolation	(37)
*R. parkeri*, Portsmouth	*A. maculatum*	Northern bobwhite quail, *Colinus virginianus*	Seroconversion	No	(37)
*R. conorii*, Malish	*Rh. sanguineus*	Dog, *Canis familiaris*	Rickettsiemia (transient); febrile illness; seroconversion	Yes (xenodiagnoses)	(38, 39)
		Hare, rabbit	Asymptomatic rickettsiemia; seroconversion	No	(40)

*^a^Name of the isolate is based on the description found in the original publication, although the identification of this isolate as R. rickettsii is very presumptive based on the biological characteristics included in the publication (26). It may, in fact, be Rickettsia montanensis but the isolate is no longer available*.

*^b^Tick species is indicated based on the known circulation of D. variabilis in the area where isolates were obtained*.

*exp., experimental animals under laboratory conditions*.

Some of these questions were independently investigated by numerous Russian investigators studying the ecology of NATT since the middle of 1930s in the vast territories of eastern and western Siberia and from eastern Altai to Primorye and the Far East of Russia (41). Like Ricketts they observed transovarial and transtadial transmission of *R. sibirica* but by different *Dermacentor* species (chiefly. *D. silvarum* and *D. nuttalli*) and by *Haemaphysalis* sp. ticks whose life cycle in turn depends upon the availability of different host animals. As one outcome of those studies, *R. sibirica* was isolated from a variety of wild rodents, including voles, susliks, lemmings, chipmunks, hamsters, striped field mice, Norway rats, house mice, and hares (41). At the time, it was postulated that under favorable climatic-landscape conditions that support specific biocenotic systems of tick vectors and host animals, natural foci of tick-borne rickettsiae can exist for many generations of ticks and host animals independent of man (42). As a part of the long-term studies conducted by Kulagin and several other investigators such as Shapiro and Korshunova et al., it was also suggested that despite being a primary animal host for larval and nymphal ticks vectoring *R. sibirica*, wild rodents are unlikely to be primary players in the maintenance of the rickettsiae [cited in Ref. (41)]. These observations were based on testing of blood and tissue samples and the conclusion that the wild rodents develop only transient rickettsiaemia and most of them did not carry rickettsiae in the spring, which is the interepidemic period for NATT. While the primary role of the tick in long-term maintenance of the agent rather than host animal reservoirs seems better established for *R. sibirica* (43), the other quantitative and qualitative questions posed above for *R. rickettsii* and other spotted fever group rickettsiae are also still unanswered for *R. sibirica*.

At present, RMSF is recognized as the most malignant of known tick-borne rickettsioses while NATT generally manifests as a relatively mild illness (44). Furthermore, *in vitro* study has also indicated a different pathogenic potential and associated capacity to cause injury among different strains of *R. rickettsii* and most of these cause much greater cellular injury than *R. sibirica* (45–47). These characteristics may also determine the rates and outcomes of rickettsial interactions with the tick vectors and animal hosts and thus determine the natural fluctuations of those cycles and persistence of the agents in the environment. Consequently, given the variables involved, it is not surprising that the incidental contact of man with these cycles can vary greatly from year to year and thus the number of cases that occur each year in a given area can fluctuate wildly.

## Associations of Tick-Borne Rickettsiae with Wild Animals and Their Sylvatic Cycles

The first isolate of *R. rickettsii* from a naturally infected animal in North America, was not made until 1954, when Gould and Miesse recovered a mild strain from the tissues of a meadow-mouse (*Microtus pennsylvanicus*) in Virginia (26). Another strain was recovered from the liver of a naturally infected cottontail rabbit (*Sylvilagus floridanus*) trapped in the same state in 1961 (28). Subsequently, isolates of *R. rickettsii* were made from spleen and liver tissues of wild mice (*Peromyscus leucopus*, *Pitymys pinetorum*), cotton rats (*Sigmodon hispidus*), a golden-mantled ground squirrel (*Citellus lateralis tescorum*), and from chipmunks (*Eutamias amoenus*) trapped in Virginia and Western Montana (27, 30). Several small animals, mainly wild rodents, were sources of *R. sibirica* isolates obtained across a large territory known for the endemicity of NATT (41).

Despite these existing field observations the question remains unanswered whether all these animals or only certain species represent efficient sources for infection of ticks and how long they can provide that function after acquiring the agent. For example, bushy-tailed woodrats (*Neotoma c. cinerea*) were consistently negative for *R. rickettsii* by isolation, although they originated from the Bitterroot Valley of Western Montana where RMSF is highly endemic (22). To address this difference, Burgdorfer et al. (22) performed quantitative analyses of susceptibility to virulent *R. rickettsii* Sawtooth for various species of small animals and evaluated their role as possible sources for infecting larval *D. andersoni* (22). The data obtained by this extensive and important study are illustrative of typical experiments but it emphasizes the limitations of laboratory experimentation. Whether the rickettsial challenge dosage vastly exceeds that delivered by low levels of repeated tick infestations and its impact where the animal may already be immune to rickettsiae, or what responses occur to infestations with single infected ticks, are important quantitative issues. Similarly, the role of tick effectors in facilitating or ablating an infection relative to intradermal, subcutaneous, intramuscular, and intraperitoneal inoculation of cultured agent is also a concern (48). It must be remembered that ticks filter large volumes of blood during their feeding so that assays of rickettsial content in samples of host blood from a single time point may not accurately reflect the amount that ticks may acquire. Wild caught animals were exposed to nymphal *D. andersoni* infected with *R. rickettsii* Sawtooth, a guinea pig virulent tick strain originating from the west side of the Bitterroot Valley (22). Columbian ground squirrels and chipmunks developed rickettsiaemias that appeared on the third or fourth day and lasted for 6–7 days with a maximum of 9 days in one chipmunk. The largest concentrations of rickettsiae in both species of rodent were found on day 6 or 7, when blood dilutions of 10^−3^ still produced infections in guinea pigs. For comparison, rickettsiaemias were observed in guinea pigs that were fed on by the same number of infected ticks as were used with the Columbian ground squirrels and chipmunks. The concentration of rickettsiae in the blood of guinea pigs was much higher, with a prolonged period of at least 6 days in which 100 or more infectious doses per 0.5 ml of blood were present. The golden-mantled ground squirrels experienced rickettsiaemia with maximum titers of at least 10^3^ for a relatively short period of time. In snowshoe hares, rickettsiaemia lasted as long as 5 days but the concentrations of rickettsiae were much lower and rarely exceeded 10 infectious guinea pig doses. Least susceptible were bushy-tailed woodrats, in which rickettsiae could be demonstrated only following attachment of hundreds of infected ticks. In meadow mice, rickettsiae circulated for as long as 6–8 days in concentrations that, in some specimens, reached at least 1000 infectious doses per 0.5 ml of blood. Subsequent experiments were conducted with Columbian and golden-mantled ground squirrels, meadow mice, and snowshoe hares. The results indicated that naive *D. andersoni* larvae that fed on these hosts during peak rickettsiaemia invariably exhibited high infection rates while those that fed during the initial or final stages ingested rickettsiae insufficient in numbers to establish permanent infection of tick tissues. Accordingly, meadow mice, Columbian ground squirrels, chipmunks, and golden-mantled ground squirrels must be considered highly efficient sources of infection, at least for those ticks that feed during the periods when large quantities of rickettsiae are present in the blood. In meadow mice, which appeared to be the most susceptible, rickettsiae circulated in concentrations of 10^2^–10^3^ guinea pig infectious doses for as long as 4 days. Similar titers, although for 1 or 2 days only, were detected in chipmunks and Columbian and golden-mantled ground squirrels. These studies demonstrated that hares do respond to infectious tick bites with rickettsiaemias that in general are much milder than those observed in ground squirrels, chipmunks, or meadow mice. However, for at least 1 or 2 days, infectious titers may reach the level necessary to infect 50% or more of larval *D. andersoni*. Despite these limitations, isolates of *R. rickettsii* were recovered from the blood of a snowshoe hare (*Lepus americanus*) (30). Finally, the bushy-tailed woodrat, a common host of immature *D. andersoni*, was the only species of animal that did not circulate rickettsiae in the blood following attachment of infected ticks, suggesting that this rodent is not susceptible to spotted fever group rickettsiae and is of no significance for infecting ticks in nature.

Although, the natural reservoir of *R. conorii* is not yet fully demonstrated, Rovery et al. suggested that rabbits could be rickettsemic without developing severe disease, so wild rabbits (*Oryctolagus cuniculus*) might be a reservoir for *R. conorii conorii* and could play a role in the transmission of *R. conorii conorii* in the French Mediterranean (40). These observations and other previous publications suggest that rabbits and hares may play a significant role in the circulation of rickettsial pathogens in nature. To some extent distribution of human cases of RMSF in the western USA coincided with distribution of Nuttall’s cottontail (*Sylvilagus nuttallii*). This animal is an important host of the larval and nymphal stages of *D. andersoni* and *H. leporispalustris*. Furthermore, there are significant overlaps in the geographic ranges of *D. variabilis* and the eastern cottontail rabbit (*S. floridans*), *D. occidentalis* and the Pacific Coast brush rabbit (*S. bachmani*) and *D*. *parumapertus* and the black-tailed jack rabbit (*Lepus californicus*). *Sylvilagus* is one of the few animal genera that is present in both North and South America, and it may have had a role in the introduction of *R. rickettsii* and other rickettsiae into South America or vice versa (5, 41). Similarly, according to Lyskovtsev, the European hare (*Lepus europeus* Pallas) was among the animals known to develop sufficient rickettsiemia to recover an isolate of *R. sibirica* (41). There is also experimental evidence suggesting a role for hares in the circulation of *R. slovaca* (49).

Many other species of wild animals have been implicated in rickettsial maintenance but solely in the context of their roles as blood meal hosts for different tick species. These animals can certainly differ in their susceptibility to rickettsial infection, whether they develop clinical disease or just subclinical infection, whether the infection is persistent or sterile immunity occurs, and the extent of their immunity to subsequent reinfection. Body size might also be important as the number of ticks that may attach can increase substantially as one progress from small to medium size to large hosts (50). Opossums have been implicated in maintenance of *R. rickettsii* in both North and South America (27, 33). Different species of opossums only develop an inapparent infection, whether in nature or after experimental inoculation, but they can sustain at least 3–4 weeks of rickettsiaemia demonstrable by tick acquisition feeding and direct isolation of rickettsiae persisting in their tissues (27, 33). Similar observations were made for the capybara (*Hydrochoerus hydrochaeris*), a large rodent, which is a primary animal host for *Amblyomma cajennense* in Brazil (31, 32).

## The Role of Dogs and Other Peridomestic Animals in Transmission and Maintenance of Spotted Fever Group Rickettsiae

Dogs are viewed as important sentinel animals for rickettsial disease agents since they can suffer clinical illness following infection with *R. rickettsii* and *R. conorii* (51). Infected dogs can present with fever, lethargy, vomiting, and anorexia, and may develop other symptoms and manifestations similar to those of the disease in humans, including ocular lesions, bleeding disorders, joint pain, and neurologic abnormalities. The factors resulting in clinical infection may include breed, other underlying health conditions, and very likely the dosage and degree of infestation of the dogs and protection arising from previous exposure to immunizing levels of ticks and rickettsial agents of low pathogenicity. Overt rickettsial diseases in dogs have been confirmed by PCR and sequencing of rickettsial DNA and seroconversion (52–54). However, many dogs are only subclinically infected and do not exhibit these severe manifestations even if they may seroconvert (55–57).

Acute MSF and RMSF in dogs are accompanied by rickettsiemia detectable between days 2 and 12 after inoculation using cell culture isolation or PCR (54), though asymptomatic dogs may remain infectious for ticks for as long as 30 days (38). These illnesses are followed by complete clearance and development of anti-rickettsial IgG; its persistence and titers depend on the number of inoculated rickettsiae (35, 57). In contrast, dogs infected with *R. montanensis*, a widely distributed SFGR of unknown pathogenicity found in *D. variabilis*, remain asymptomatic (58). However, such exposure is usually sufficient to elicit a cross-protective immune response to subsequent inoculation with *R. rickettsii* (58). Antibody responses in dogs infected with *R. rickettsii* show a similar pattern of reactivity to *R. rickettsii*, *R. montanensis*, *R. rhipicephali*, and *R. bellii* (59). However, treatment with tetracycline causes significant delays in serologic responses of infected dogs to heterologous rickettsial species (59); as in humans, untreated canine rickettsial infections may result in fatalities (60). Several case reports in the literature describe diagnosis of RMSF in dogs associated with and, in some cases, leading to identification of the infection in people in the same household or vicinity (61, 62). Rickettsiae are transmitted by ticks, rather than directly from one infected dog to another. Manual removal of engorged ticks from dogs has been identified as a potential risk factor for human infection, which can occur by self-inoculation of the pathogen onto mucous membranes by contaminated fingers as has been shown with *R. conorii* and *R. conorii caspiae* (63, 64).

Dogs also play an important role as biological hosts of several tick species, which can transmit rickettsiae to humans and other dogs (51). Once infested, dogs serve to increase the infected tick populations present in close association with human habitats, and can introduce infected ticks into the peridomestic environment (65). In the case of *R. conorii*, laboratory experiments demonstrated that dogs are a competent reservoir for this rickettsia (38); whether dogs become rickettsemic with every human or guinea pig-pathogenic *Rickettsia* is not known. In an experimental setting, beagles subcutaneously inoculated with *R. japonica* did not produce rickettsiemia or clinical symptoms of infection (56). Dogs with different genetic backgrounds appear to differ in their susceptibility to rickettsiae and *R. conorii* infection in particular (38).

In the new world, the association of *Rh. sanguineus* with dogs acquired new importance and attention after the rediscovery of a sustained transmission cycle of *R. rickettsii* by this tick in arid regions of North America. The first site was discovered by recognition of atypical foci of RMSF in eastern Arizona well outside the distribution of *Dermacentor* sp. ticks (66). Similar foci were subsequently discovered in Brazil and several sites in Mexico (67, 68). The genotype of *Rickettsia rickettsii* circulating in AZ has a unique genotype that differs from those of *R. rickettsii* in Mexico and Brazil (23, 68). Furthermore, the brown dog ticks in Brazil and Mexico associated with those outbreaks differed from those in the Arizona outbreak (68). In either region, dogs are considered to be an amplifier of rickettsial prevalence through co-feeding transmission by the infected ticks. Surprisingly, *R. rickettsii* from South America does not cause any substantial mortality in the infected brown dog ticks and exhibits more efficient transstadial and transovarial transmission than that occurring in USA (34). These outcomes are quite different from the interactions of *R. rickettsii* and *Dermacentor* ticks in Northern America that are discussed below (69).

Another human pathogen found in *Rh. sanguineus* is *R. massiliae* (70). It was originally described in *Rhipicephalus turanicus* from France, but has since been identified in several other countries including the new world (1). One genotype of *R. massiliae*, known as Bar29, has been shown to infect humans (70, 71). The USA genotype AZT80 (of Bar29 genotype) of *R. massiliae* was implicated as a cause of canine illness in California; however, those associations could not be confirmed beyond serological observations (72). Additional work is needed to define the importance of *R. massiliae* in human and animal health. Likewise, further work will be required to validate observations associated with the natural exposure of dogs to various SFG rickettsiae through tick bites (73).

Other species of spotted fever group rickettsiae have been identified in ticks that bite both dogs and humans so that dogs may play some role in their eco-epidemiology. In most cases, only laboratory evidence for canine susceptibility to infection has been obtained and their role as a source of human infection is less certain. These studies are most advanced with *Rickettsia parkeri*, which is long known from tick surveys but which has only recently been recognized as an emerging pathogen of humans in USA and in Uruguay, Argentina and Brazil (1, 73–75). In Brazil, dogs are commonly infested with *A. ovale* and *A. aureolatum*, which frequently carry the *R. parkeri*-like Atlantic Rain Forest *Rickettsia* (73). Similar agents have been found in other *Amblyomma* species from birds (*A. calcaratum*), capybara (*A. dubitatum*), anteaters (*A. nodosum*), marsh deer (*A. triste*), dogs (*A. tigrinum*), and *A. maculatum* from dogs, horses, and cattle in Peru (76–81). Whether all these variants can cause human disease is unknown but the large number of tick species containing *R. parkeri*-like agents and their diversity of hosts suggests that understanding their maintenance and transmission will be challenging. However, the *A. triste* agent could be maintained with high efficiency for five generations of ticks on rabbits by transovarial and transstadial passage; this agent is thought to be a primary human disease agent in the southern countries of South America and it is very similar to North and South American strains from *A. maculatum* (80). However, the causation and significance of the apparent bimodal distribution of agent load in the *A. triste* ticks is unclear. Cattle have been recognized as hosts for *A. maculatum* for many years in USA (36). Laboratory studies conducted in Mississippi indicate that upon exposure to *R. parkeri* by injection or by feeding *R. parkeri*-infected *A. maculatum* calves seroconvert to *R. parkeri* antigen, but only a few animals develop short-lasting rickettsiemia (36). In a parallel field study, cattle were not rickettsiemic, suggesting that they only play a critical role in tick feeding and vagility, and thus in maintenance of *R. parkeri* by vertical and transtadial transmission. However, the ticks also appear to stimulate rickettsial growth in host tissues during engorgement (48). Migrating birds may have an important role in dissemination of *R parkeri* and other agents present in ticks including their importation between the continents (82).

The role of cats in the eco-epidemiology of tick-borne SFG rickettsioses has received much less attention than evaluations in dogs because cats are less frequently infested with ticks. Typically <10% of free ranging cats have ticks, but some may be infested with large numbers of ticks (83). Moreover, serological findings of rickettsial exposures in cats must also be carefully interpreted due to possible cross-reactivity with the much more widely prevalent flea-borne agent, *R. felis*, found commonly in cat fleas and frequently in other flea species (84). Detection of DNA from *R. massiliae* and other unidentified other core spotted fever group rickettsiae in ticks on cats indicates that cats may serve as an important peridomestic source of infection in some situations (85).

The importance of African ruminants in natural cycles of many tick-borne agents like those causing heartwater and bovine anaplasmosis has been very well documented. Less clear is the role of domestic stock in maintenance of rickettsial agents in peridomestic settings where they might directly cause human disease. Molecular surveillance data reported by Mutai et al. (86) implied that substantial and comparable numbers of Kenyan domestic animals develop rickettsiemia (based on quantitative PCR detection of the conserved rickettsial 17 kDa protein gene fragment): 16.3% in cattle and 15.1% in sheep, but only 7.1% in goats, which were also less frequently infested with ticks (86). However, because the ticks were collected directly from the animals, one cannot know if they were transmitting or acquiring rickettsial agents. Four known human pathogenic species were detected: *R. africae* was detected in 93% of PCR positive ticks (seven species, four genera including *Amblyomma*, *Hyalomma*, *Rhipicephalus*, and *Boophilus*), while *R. mongolitimonae*, *R. aeschlimannii*, and *R. conorii israelensis* were found infrequently in addition to several other less-well known genotypes of *Rickettsia*. In Israeli, *Hyalomma* ticks from camels as well as several camel bloods were infected with *R. aeschlimannii*; *R. africae* was also detected in all four species tested (87). Similarly, in Egypt, *Hyalomma dromedary*, *H. impeltatum*, and *H. marginatum marginatum* collected from camels in some areas had high rates of rickettsial infection with *R. africae* (over 57% of *H. dromedary*) and *R. aeshlimannii* (over 73% of *H. impeltatum*) (88). Determining the frequency which these ticks bite humans as well as their interactions with and dependence on other potential wildlife reservoirs of these rickettsiae will be required to determine their significance for public health.

## Effects of Spotted Fever Group Rickettsiae on Ticks

**Figure 1:**
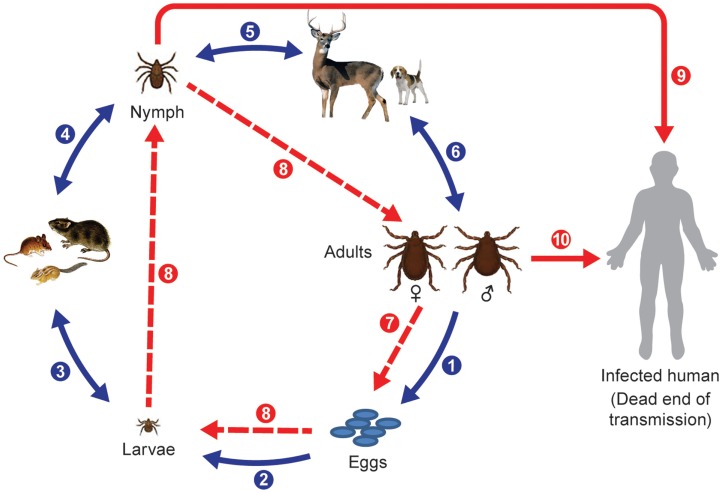
**Life cycle of Ixodid ticks and natural transmission of rickettsiae**. Blue arrows indicate main steps of tick natural cycle: (1) oviposition by engorged female; (2) eggs hatched into larvae; (3) larvae feed on small animals; (4) engorged larvae hatch into nymphs; (5) nymphs feed on large or small animals; and (6) nymphs molt into adult ticks that feed on large animals or bite humans. Broken red arrows indicate transovarial (7) and transstadial transmission (8) of rickettsiae, and solid red arrows indicate transmission of rickettsiae to humans through a bite of a nymph (9) or an adult tick (10).

Hard ticks (Ixodidae) are the primary vectors of spotted fever group rickettsiae and they develop through three discrete life stages (Figure 1). However, recently soft ticks (Argasidae) have also been found to harbor SFG rickettsiae of unknown pathogenicity (89); they typically feed rapidly and thus do not stay attached for long periods and do not have a scutellum or the pronounced morphological differentiation found after molting from larvae to nymphs to adults as occurs in hard ticks. The natural life span of non-nidiculous ticks and the survival and expansion of tick populations depends on the degree of blood satiation and environmental factors, particularly, temperature and humidity and habitat types and host abundance; therefore, changes in annual seasonal conditions appears to be more important for the success of some temperate zone ticks species than for others (90–94).

Ticks can have one host, two host, or three host life cycles. Two-host ticks feed as larvae and nymphs on the same host. Following detachment and dropping to the ground or leaf litter after blood engorgement on a host animal, the fertilized female deposits eggs in sheltered places in crevices of the soil surface or grass. The fertility of individual females depends on the tick species and degree of engorgement, so the numbers of eggs laid vary from 3000 to 8000 per female as estimated for *Dermacentor* ticks [cited in Ref. (41)]. Larvae hatch between day 4 and 82 after oviposition and quest very close to the original egg mass; they molt into nymphs after they have obtained a blood meal. Larvae and nymphs of *Dermacentor*, *Haemaphysalis*, and *Amblyomma* sp. ticks feed on small mammals, insectivores, rodents, small carnivores, and birds. *Haemaphysalis* spp. also parasitize wild birds. Engorged nymphs detach from the hosts and molt into adult ticks in 11–25 days. Adult ticks parasitize large wild and domestic animals. In contrast, one host ticks like *Rhipicephalus* sp. and *Amblyomma albipictus* differ from many other Ixodidae by feeding on a single animal species; their different stages can be found frequently at the same time on an infested host. Nymphs and adults will attach and feed on animals long enough to transmit rickettsiae if infected (95). Alternatively, as has been mentioned previously, larvae and nymphs can become infected by feeding on a rickettsiemic animal, and frequently and most efficiently nymphs and adults can become infected through co-feeding transmission of agents from other tick stages (96, 97). Aggregation of ticks on hosts appears to be essential for keeping the infection rates of ticks at environmentally sustainable levels (98). This mechanism can operate in the face of host immunity so it is a very important mechanism for rickettsial maintenance in nature, especially when ticks are abundant (39, 99). Humans and other hosts can become accidental victims of exposure to rickettsia-infected ticks, and can suffer from febrile disease or become rickettsiemic and infect feeding ticks but this acqusition route probably does not contribute significantly to the maintenance of rickettsiae in nature compared to transovarial, transtadial, and co-feeding mechanisms.

For most SFGR, the transovarial-transtadial pathways appear to be the essential mechanisms for their maintenance in the environment because this occurs independently of tick density and can cause significant expansions in the infected tick populations just from the progeny of single ticks; however, the molecular mechanism(s) of pathogen host tolerance and potential mutualism are not at all well understood even if many pathogen genomes are completely sequenced. The host–agent interactions (or relationships) apparently do vary substantially among rickettsiae with differing pathogenic potential toward humans and animals, possibly accounting for the large numbers of species of ticks and hosts that harbor agents and for their variable responses to the presence of those agents. The diversity of interactions is certainly very clear experimentally with respect to the effects of different agents on the ticks themselves. Two very different scenarios are evident but their relative importance and degree of parallel or synergistic occurrence varies with the agent and the tick host. In the first extreme, transovarial transmission is regarded as the sole mechanism of maintenance of the rickettsial agent in a given population of ticks. This is most evident for the rickettsial “obligate endosymbionts,” which have not been cultivated outside of ticks except in continuous tick cell lines (100). Rates of 100% infection are achieved in species like *I. scapularis* and *I. pacificus* but the agent appears not to be transmissible to other hosts. The second extreme is where the *Rickettsia* agent can have variable effects on the tick host and is generally acquired by uninfected ticks from persistently infected vertebrate hosts or must be acquired by co-feeding from ticks, which have already acquired those agents. In *D. andersoni* ticks infested with *R. rickettsii*, the rate of transovarial transmission was estimated to be from 35 to 100% by mild or massively infected females, respectively (101, 102) while it appears to be close to 100% in *Amblyomma americanum* with *R. amblyommii* (14). Independently, this mechanism was established and recognized as a significant part of transmission and natural maintenance of *R. sibirica* in *D. nuttalli* by S. M. Kulagin [cited in Ref. (41)] and *R. conorii* (103). Subsequently, inheritance of a pathogen was found to depend on multiple variables associated with the experimental conditions (104). The importance of these factors on natural maintenance of *R. rickettsii* is less clear. In fact, a pronounced detrimental effect of the Sawtooth strain of *R. rickettsii* on survival and oviposition rates of *D. andersoni* females as well as their fecundity was observed in experiments conducted with laboratory reared ticks (69). Similarly, AZ-type highly virulent isolates of *R. rickettsii* appear to have a detrimental effect on *Rh. sanguineus* circulating in Arizona, which was determined by detection of the decreased prevalence of *R. rickettsii* in larvae and nymphs developed from eggs laid by infected females (unpublished personal observation) and variable rates of transovarial transmission and filial infection rates with *R. rickettsii* were seen between infected *A. cajennense* and *A. aureolatum* (105). Interestingly, this is not a unique association since *R. conorii* Malish exhibited similar effects on *Rh. sanguineus* ticks (106–109). However, these observations on adverse effects of highly virulent spotted fever group rickettsiae are probably not universal and depend on many yet to be identified variables (Table 2). No substantial mortality difference was observed between uninfected *Rh. sanguineus* or following infection with a Brazilian strain of *R. rickettsii* (34); similarly, there was minimal cost due to the acquisition of *R. massiliae* by *Rh. turanicus* (110). While the subspecies *R. conorii israelensis* and *R. conorii* Malish are closely related, their biological differences in terms of their effects on tick survival and ability to maintain the agent transovarially and transtadially were dramatically different (108, 109). Observations with *H. leporispalustris* ticks infected with *R. rickettsii* Taiaçu strain suggested an increased fitness of the infected ticks (111), a result analogous to the positive role of endosymbionts in many arthropod hosts. In general, it appears that the rate of transovarial transmission and extent of damage caused in a particular tick-host system depends upon the particular strain and species of *Rickettsia* being used and how well it propagates in the ovary of a particular tick.

**Table 2:** **Effects of spotted fever group rickettsiae on their tick vectors**.

*Rickettsia* species, isolate	Tick species (origin)	Effects	Reference
*R. rickettsii*, Sawtooth	*D. andersoni* (Montana)	↓Larval and nymphal molting	(69)
		↓Female feeding success	
		↓Oviposition	
		↓Reduced transmission of rickettsiae	
*R. rickettsii*, Como-96	*D. andersoni* (Montana)	↓Larval and nymphal molting	(69)
		↓Female feeding success	
		↓Reduced transmission of rickettsiae	
*R. rickettsii*, Wachsmuth	*D. andersoni* (Montana)	↓Larval and nymphal molting	(69)
		↓Female feeding success	
		↓Reduced transmission of rickettsiae	
*R. rickettsii*, Taiaçu	*A. aureolatum* (Atibaia, Saõ Paulo)	↓Larval and nymphal molting	(112)
		↓Oviposition	
*R. rickettsii*, Taiaçu	*A. cajennense*	↓Transovarial transmission	(105)
		↓Reproductive performance	
*R. rickettsii*, Taiaçu	*Rh. sanguineus* (Seropédica, Rio de Janeiro)	Low filial infection rate (<50%)	(34)
		Low larva infection rate (7.8–8.3%)	
*R. rickettsii*, Taiaçu	*Haemaphysalis leporispalustris*	↑Biological performance	(111)
*R. peacockii*, Skalkaho	*D. andersoni* (Montana)	No effects	(69)
*R. conorii*, Malish (VR163)	*Rh. sanguineus* (Thailand)	Detrimental effect	(107)
*R. conorii conorii* (strain not identified)	*Rh. sanguineus* (Southern France)	↓Molting success	(113)
		↓Longevity of nymphs	
		↓Infection rate in survived ticks	
		↑Mortality	
*R. conorii conorii*, Ghazonet	*Rh. sanguineus* (Algeria)	No detrimental effect observed	(114)
		100% Transovarial transmission	
		99% Filial infection rate	
*R. conorii conorii*, Malish (VR163)	*Rh. sanguineus* (North American and Mediterranean colonies)	Significant effect observed	(108)
*R. conorii israelensis*, ISTT-CDC1	*Rh. sanguineus* (North American and Mediterranean colonies)	No significant effect observed	(108, 109)
*R. massiliae*, Bar 29	*Rh. turanicus* (Corsica)	No detrimental effects observed	(110)
*Rickettsia africae*, ESF 2500-1	*Amblyomma variegatum*, (Ivory Cost)	No detrimental effects observed	(115)
		100% Transovarial transmission	
		93.4% Filial infection rate	
*R. montanensis* (strain not identified)	*D. variabilis* (Old Dominion University colony)	No detrimental effects observed	(116)
*R. montanensis*, M/5-6	*D. andersoni* (Montana)	↓Rate of transovarial transmission	(69)
*R. rhipicephali* (strain not identified)	*D. variabilis* (Old Dominion University colony)	↓Egg mass weight	(116)
		↓Rate of transovarial transmission	

Transovarial maintenance of *R. peacockii* (East Side Agent) within the maternal lineage of *D. andersoni* has been hypothesized to result in a reduced probability of acquisition and transovarial transmission of *R. rickettsii* (104, 117) – the so-called interference phenomenon between these microorganisms although the data supporting the hypothesis have been criticized (104). The molecular mechanism(s) of this interaction is unknown, but in the experiments that served as a base for this hypothesis the ticks whose ovarial tissues and deposited eggs contained *R. peacockii* acquired *R. rickettsii* less efficiently from guinea pigs than uninfected ticks. The ovaries containing *R. peacockii* also excluded *R. rickettsii*, which could grow in other tissues in the tick. It appears, however, that infected ticks may acquire a second and even a third rickettsia through a bloodmeal (118, 119). Exclusion of *R. rickettsii* by *D. andersoni* previously infected with either *R. rhipicephali* or *R. montanensis* also appears to occur (117). Capillary fed *D. variabilis* infected with either *R. rhipicephali* or *R. montanensis* demonstrated mutual exclusion and lack of transovarial transmission of the superinfecting rickettsia (116). These processes may be regulated by differential expression of tissue-specific, and in some cases *Rickettsia* species-specific, selected tick immune genes as demonstrated in experiments evaluating the response of *D. variabilis* to *R. amblyommii* and *R. montanensis*, respectively, using an *ex vivo* tick organ model (120). The ecological relevance of these incomplete experiments is suggested by the limited number of cases of RMSF, which occur in areas where *R. peacockii* is prevalent compared to similar geographic areas where it is much less prevalent. However, whether this observation is applicable to other ticks and rickettsial agents and whether the fundamental mechanisms involved are conserved will need substantial study. The presence of *R. bellii* in *Amblyomma dubitatum* appeared to reduce the acquisition of *R. rickettsii* from rickettsemic animals but transmission still occurred in some ticks (121).

## Modeling of Tick-Borne Rickettsial Diseases

Prediction of the transmission dynamics of zoonotic and vector-borne diseases that are associated with wildlife present many challenges due to the absence or insufficient characterization of wildlife host species, pathogens, and vectors in many locations. The null case for tick-borne rickettsioses is simple in that the vectors themselves have an essential role(s) since transmission can only rarely occur in their absence. In other words, direct transmission from infected animal reservoirs, the definition of a zoonosis, is actually rare or lacking altogether in the absence of vectors. Similarly, the incidence of human rickettsioses is also nearly zero when their lifestyles and activities do not bring individuals into any contact with animals or vectors. However, the range of variables to consider and measure beyond these two extremes makes useful modeling a daunting task.

Several aspects of the potential impact of ticks on human health have been evaluated in recent years. Particularly, satellite data coupled with sophisticated Geographic Information Systems (GIS) have permitted the evaluation of the relationship of various parameters such as occurrence and distribution data on ticks to defined ecological niches and animal host ranges, and the extrapolation of climate change data to future risk assessments (122). The basic starting concept is that each species of tick and its animal hosts are found within specific ranges of environmental variables (temperature, humidity, ecotypes), which support their reproduction and individual survival (123), so that the existing climate and environmental parameters associated with an agent define a set of conditions necessary for the predicted existence of a particular population in a very specific small area or averaged over a much larger region (122). The geographical range of a tick population depends on many parameters ranging from the life cycle of the tick, abundance of its hosts, and anthropogenic influences on vegetation, land use, and host displacement (124, 125). The Mediterranean region is expected to experience the greatest changes in risk of tick-borne infections in Europe due to predicted increases in average temperature and decreases in average rainfall. The climatic changes are predicted to affect the distribution of several tick species including expansion of ranges for *Rh. turanicus* and *Hyalomma marginatum marginatum*, and retreats for *D. marginatus* and *Rh. bursa*, which will be displaced toward higher latitudes (122, 126). Climate-based modeling conducted for human biting *D. andersoni* indicated a shift toward peak abundances of *D. andersoni* adults occurring in sheltered northern/eastern exposures, rather than in drier and hotter southern/western exposures (127). Modeling of the impacts of the changes in the climatic conditions in France on the activity and distribution of *Rh. sanguineus* confirmed empirical observations of the northward migration trend of this cosmopolitan tick, which carries many human and veterinary pathogens (128). Historic changes in the geographical distributions of ticks within different parts of their ranges have already been observed for several species of Ixodid ticks such as *Ixodes ricinus* in northern European countries (129) or *Amblyomma americanum* in USA (92). In this regard, meteorological data and weather forecasts appear to be useful for predicting the activity and density of ticks, particularly as was implemented for predicting infections transmitted across Europe by *I. ricinus*, *D. reticulatus*, *Rh. sanguineus*, and the flea *Ctenocephalides felis* (130). Maps were constructed (www.FleaTickRisk.com) that use current meteorological data for weekly predictions of ectoparasite activity in different areas. The activity index of the previous week is used as the criterion for estimating the risk of tick infestation and associated transmission of several tick-borne pathogens, including *Rickettsia* for the coming week. The data supplied by the model are used as an epidemiological tool by veterinarians and other healthcare professionals to improve the advice they provide to pet owners, but this approach may serve as a good model for developing similar efforts to forecast the risk of human tick-borne diseases.

At a finer scale, measurement of the annual changes in the population size and density of wildlife hosts and different contributions of various host species to tick success may also help to predict the persistence and transmission frequency of a given tick-borne pathogen, and thus its potential for spillover at the interface of human and wildlife habitats (131). Because of the complexity of the variables, only a few attempts have been made to model local aspects of tick-borne rickettsioses as opposed to the wider impact of meteorological factors on the distribution of the host ticks. An attempt to understand the spatial concordance between RMSF incidence and the habitat probability of its main vector *D. variabilis* is perhaps the best example of the value and limits of this approach (132). The latter study specifically focused on defining these habitat associations only in parts of Texas, but it was clearly limited by the small amount of data available at the site including insufficient tick sampling, unsophisticated diagnosis of human SFG rickettioses, an ineffective reporting system for RMSF, and anthropogenic inferences due to movement of people and various economic and agriculture activities, which directly affected tick habitats during the study period.

Earlier models of *R. rickettsii* transmission in *D. andersoni* and *D. variabilis* had been done without our current understanding of both the biology of rickettsia-tick interactions and the contemporary view of the molecular epidemiology and biogeography of RMSF (4, 24). Consequently, the original predictions derived in those studies do not meet current expectations but could serve as the basis for an updated model. Early modeling of *R. rickettsii* transmission in *D. andersoni* (19) analyzed only the role of vertical transmission in maintaining *R. rickettsii* infection and its potential effect on the size of the vector population, particularly the cumulative effect of a pathogen load passed down through consecutive generations based on a varying 10–100% transmission rate (102). The load of *Rickettsia* in the population would increase with each successive generation if it were predominantly vertically acquired; its accumulation might then eventually become a factor in controlling the tick population size and indirectly affect the survival of the host because of the damage caused by this organism.

Limited later modeling attempts, which considered additional variables for both vector and pathogen, established the following predictions. Simulated *in silico* predictions based on estimated relationships between the rate of transmission and the density of ticks determined that approximately 252 adult *D. variabilis* per ha are required to sustain transmission of *R. rickettsii* (133). These calculations were based on the assumption that a maximum of 98% of engorged immature nymphs of *D. variabilis* survive to the adult stage (134), and took into consideration the various effects of biotic and environmental variables including weather, host density and their habitat, infectivity levels of ticks and mammals, as well as the fecundity of infected ticks and the efficiency of transovarial transmission of rickettsiae (133). The authors emphasized the deviations between reported and simulated cases in Maryland and Oklahoma (133), but were unaware of the predominant presence of *R. montanensis* in *D. variabilis* in Maryland and elsewhere in USA (135, 136). The same estimates of tick densities were used to test if the occurrence of RMSF can be predicted based only on the presence of particular mammalian species as well as the relative abundance of important host species and their effect on the adult *D. variabilis* population size (137).

Cooksey et al. defined the RMSF transmission threshold as the density of ticks at which the yearly rate of increase of rickettsiae to humans is at an equilibrium level or 1.00 (133). It was estimated that for the RMSF potential of 1.61 to occur required the availability of 102 immature *D. variabilis* per 0.4 Ha, which with a 98% survival rate results in 252 adult ticks in the area (133, 137). The most accurate estimates were predicted for *D. variabilis* infesting raccoons and opossums with infestation rates ranging from 0 to 17; three sites were predicted to have a RMSF potential of >1.61. These estimates were then the state of the art based on the existing modeling approaches and the available understanding of RMSF ecology and epidemiology; the current information based on molecular epidemiology data makes those estimates very questionable. The state of Tennessee is ranked among the areas with the highest reported rate of RMSF and highest morbidity and mortality (138); however, field studies and large scale testing of ticks failed to even demonstrate the presence of *R. rickettsii* in any of the associated territories (139). Instead, only a high prevalence of *R. montanensis* was detected.

For *D. variabilis*, the presence and numbers of immature ticks seem to be the most important determinants defining the dynamics of various species of *Rickettsia*. Thus, it seems crucial to evaluate the actual burden of ticks on the host animal populations to accurately measure this variable. To address these questions, Dallas et al. (124) evaluated the association of nymphal and larval *D. variabilis* with its primary mammal host, *P. leucopus* and computed the tick burden in relation to other host variables, including mass, sex, and habitat (124). Consistent with other rodent-tick systems, this study demonstrated that the burden of immature *D. variabilis* is positively associated with male mice of higher body mass captured in the field habitat (124). This correlation is likely due to the higher probability of males to encounter ticks due to their larger home range and higher susceptibility to tick infestations because of their higher surface area.

Static models of tick-borne diseases are obviously limited due to their cross-sectional character and limited assumptions based on measurable parameters. Dynamic models would appear to encompass more of the essential information required to predict and evaluate other parameters like invasion of tick species with their associated tick-borne pathogens as might occur with exotic ticks on birds (82). While focused on transmission of the agent of human monocytic ehrlichiosis, *Ehrlichia chaffeensis*, which must be acquired by ticks every generation since it is not maintained transovarially, a broader agent-based model for tick–borne disease(s) was recently reported (20). It evaluates the interactions between ticks and their hosts as well as the transmission of tick-borne disease between two populations. The applicability of this model to rickettsial diseases has not been tested. However, the model predicts a significantly lower prevalence of ehrlichiae in both ticks and their hosts compared to predictions made with similar data using other models.

## Critical Questions and Future Directions

As we have discussed, the distribution and prevalence of tick-borne pathogens and the diseases they transmit are strongly influenced by many factors, including changes in broad geographic and local climatic parameters, variations in land use, changing human activities, and animal behaviors that may cause disruption of ecosystems. The accuracy of the estimates of human disease depend greatly upon the adequacy of contemporary medical and public health practices including the extent and efficiency of surveillance efforts and the prevalence and specificity and quality of diagnostic practices in clinical practice. Changes in landscape ecology may result in the pronounced expansion of the number of ticks or of their biological hosts, with a consequent increased risk for human or animal health. In contrast, human activity can cause pronounced habitat fragmentation and associated alterations in the movement of hosts carrying ticks. Those movements may also affect the dynamics of disease transmission due to alterations in the biodiversity of the hosts, vectors, and pathogens present in “island” habitats. In the context of rickettsial diseases, those juxtaposed changes have resulted frequently in the detection and description of emerging and reemerging rickettsioses both in endemic settings and globally (1), increased recognition of travel associated rickettsioses (140), discovery of previously unknown pathogenic agents (141), and the belated recognition of the overlapping circulation of several rather unrelated bacterial agents that, nonetheless, may share the same tick vectors and influence the transmission of those pathogenic for vertebrates (142).

In recent years, because of enhanced concerns over global warming, much attention has been paid to the effects of climate change on arthropods. While it is clear that many tick-borne diseases are experiencing apparent increases, which factor is the key causative variable associated with this increase is less clear. In USA, northward and western expansion of the distribution of *A. americanum* and inland distribution of populations of *A. maculatum* has created new areas for exposure to the several tick-borne diseases transmitted by these ticks (92, 143). Similarly, the continued northward movement of *Rh. sanguineus* in Mediterranean countries and presence at higher elevations of *Dermacentor* ticks in European countries (122) has increased concerns for transmission of diseases in those areas. However, while straightforward, the environmental sampling of ticks and screening for different pathogens is necessarily very sporadic and lacks an associated systematic assessment of tick density and whether different animal reservoirs and changes in habitat may have become important in those sites. This combination of factors has made it very difficult to model the risk of infection under the influence of novel or unquantified variable host factors, including the diversity of small and large animal hosts, their varying competence as reservoirs for pathogens, and the interplay between known and novel undescribed pathogens. Although this information might be interpreted in the context of fairly well understood spatial and temporal patterns of tick distributions, the microspatial factors, and seasonal fluctuations can greatly influence the focal presence and geography of ticks and their interaction with multiple animal hosts and their associated microbiota.

Ticks do not simply serve as an environment for rickettsial propagation and maintenance through the germ line. Instead, species of *Rickettsia* exhibit a continuum of interactions with their arthropod hosts. While some may act as the prototypical vertically maintained endosymbiont with potential benefit to the tick, others are opportunistic pathogens or transient commensals employing vertical and horizontal transmission mechanisms to varying degrees but without obvious or yet known effect on the tick, and fortunately for tick populations, only a select few cause major detrimental effects on their tick hosts. However, the molecular factors that tip the balance between these life styles for different species, and even strains of *Rickettsia*, are not yet understood even though the genome sequences of many of these isolates have been obtained. The rapid use of other advanced molecular tools in microbiome surveys have further revealed the complexity of the microbial communities associated with different tick species. These can include other bacteria, viruses, protozooans, and fungi, each with their own biology, and potential effects on host–pathogen interactions and diverse interactions with the vectors that harbor them (144–146). The quantitative ratio of rickettsial pathogens to the total microbial community in ticks may vary significantly depending on the tick species, its life stage, and the sex of the adult (147). Much needs to be learned about the functional, and potentially genetic, interactions between different microbes in ticks and the effects of the quantity of different microbial taxa on the tick. Whether intrinsic symbiont populations play a role in the selective acquisition, transmission, and expression of virulence factors by rickettsial pathogens needs further investigation. Whether those interactions can be exploited for control of the vector or for blocking acquisition of the pathogens are important practical and public health issues. Both systematic environmental sampling to assess the consistency of certain components of the bacterial community and state-of-the-art laboratory manipulations will be required to dissect both their natural ecological importance and their potential practical applications (148).

One of the most profound developments of the last decade in the eco-epidemiology of tick-borne rickettsioses has been its increasing numerical impact on public health. Sporadic disease certainly results from the complex and multifaceted interactions occurring between human and reservoirs of infected ticks sustained by wildlife. However, increasingly larger outbreaks of some tick-borne diseases are mediated directly through domestic and companion animals, which harbor large populations of infected ticks. One of the best examples is the persisting foci of *R. rickettsii* associated with *Rh. sanguineus* and peridomestic dogs in eastern Arizona and sites in Mexico, which serve both as hosts for feeding the ticks, the “reservoir” for these rickettsiae, and the site for co-feeding transmission of the agent (66, 68). Although the exact origin of the agents causing these sustained outbreaks is not fully understood, they arose independently because of the difference in the genotype of both the tick and the *Rickettsia*. The foci also embody good examples of how human-aided movement and maintenance of dogs can sustain the propagation of their ticks and their rickettsiae and the potential for their rapid spread into new environments. Even sustained efforts to decrease tick populations with acaricides were unsuccessful while elimination of the dog host immediately reduced transmission.

Other Ixodid ticks, which have adapted to two or three vertebrate host cycles, have significant opportunities for expanding the range of some rickettsioses to very large territories when the infected ticks migrate with passerine birds as immatures (149). Increasing numbers of the reports have described the presence of known and novel rickettsiae and other tick-borne pathogens in ticks collected from migratory birds (82, 150). Birds can serve not only as a larval tick host and vehicle for their migration for very long distances but can also establish persisting rickettsiemia [cited in Ref. (41)] that provides an infected host for different ticks at the end of a migration. In this manner, birds can serve as a highly mobile dispersive large effective reservoir for rickettsial pathogens. Establishment of new endemic foci at sites during the migration routes does require an optimal combination of climate and ecological factors and the availability of susceptible ticks and competent animal hosts to facilitate further dispersal and maintenance of the rickettsiae and for human exposure to these agents. Although many of these interactions have been postulated, systematic and quantitative assessment of these importations need to be conducted as well as broadened studies of the vector competence of these ticks and the susceptibility of potential native animal hosts to the imported pathogens.

## Conflict of Interest Statement

The authors declare that the research was conducted in the absence of any commercial or financial relationships that could be construed as a potential conflict of interest.
